# Collapsibility of jugular veins, subclavian veins and inferior vena cava as predictors of fluid responsiveness in patients on pressure support ventilation: a prospective cohort study

**DOI:** 10.1186/cc14264

**Published:** 2015-03-16

**Authors:** Y Iizuka, M Sanui, T Nomura

**Affiliations:** 1 Jichi Medical University Saitama Medical Center, Saitama, Japan; 2 Shonan Kamakura General Hospital, Kamakura, Japan

## Introduction

The accuracy of predicting fluid responsiveness (FR) using IVC collapsibility is high in patients on controlled mechanical ventilation, but remains unknown in spontaneously breathing patients with mechanical ventilation. Also, adequate ultrasound images of IVC are difficult to obtain in a substantial number of patients. The aim of the current study is to evaluate utility of collapsibility of jugular veins (IJV) and subclavian veins (SCV) in comparison with collapsibility of IVC in patients on pressure support ventilation.

## Methods

Patients on pressure support ventilation were prospectively included when fluid challenges were clinically indicated. Bilateral IJV were examined at the level of cricoid cartilage. Bilateral SCV were measured where the veins crossed the clavicle. Anteroposterior diameter, cross-sectional area (CSA) of IJV and SCV were measured using frame by frame analysis. IVC was measured 2 cm from the right atrial border in a long axis view. Fluid responsiveness was defined as 8% increase of stroke volume calculated by the Vigileo monitor (Vigileo, FloTrac; Edwards Lifesciences) after passive leg raising (started from supine position). Receiver operating characteristic (ROC) curves were generated using EZR.

## Results

Twenty-nine patients (35 measurements) were included. Nineteen measurements had fluid responsiveness. The mean tidal volume was 9.8 ml/predicted body weight. The area under the ROC curve of IVC collapsibility was 0.576 (95% confidence interval (CI): 0.38 to 0.77), while the area under the ROC curves of right IJV, left IJV, right SCV and left SCV collapsibility were 0.870 (95% CI: 074 to 1.0), 0.54 (95% CI: 0.34 to 0.74), 0.62 (95% CI: 0.43 to 0.81) and 0.54 (95% CI: 0.34 to 0.74), respectively. Greater than 11% of right jugular vein collapsibility predicted fluid responsiveness with a sensitivity of 79% and a specificity of 94%.

## Conclusion

Our results suggest collapsibility of the right jugular vein can be a useful predictor of fluid responsiveness in patients on pressure support ventilation, compared with other central large veins. Collapsibility of IVC does not predict FR in those patients.

